# A socioenvironmental approach to the nosogenic potential of freshwaters with presence of thermotolerant free-living amoebae in Costa Rica

**DOI:** 10.3389/fpubh.2025.1675182

**Published:** 2025-10-06

**Authors:** Johan Alvarado-Ocampo, Juan José Romero Zúñiga, Julián Castro, Frida Chaves Monge, Marco Ruiz Campos, Alexa Bustamante Cortés, Elizabeth Abrahams Sandí, Lissette Retana Moreira

**Affiliations:** ^1^Departamento de Parasitología, Facultad de Microbiología, Universidad de Costa Rica, San José, Costa Rica; ^2^Centro de Investigación en Enfermedades Tropicales (CIET), Universidad de Costa Rica, San José, Costa Rica; ^3^Programa de Investigación en Medicina Poblacional, Escuela de Medicina Veterinaria, Universidad Nacional, Heredia, Costa Rica; ^4^Instituto Clodomiro Picado, Facultad de Microbiología, Universidad de Costa Rica, San José, Costa Rica

**Keywords:** *Naegleria*, freshwater, sediment, risk, recreation, behavior, physicochemical

## Abstract

**Introduction:**

Within the group of free-living amoebae (FLA), the genus *Naegleria* stands out for including species adapted to high temperatures, such as the facultative human parasite *Naegleria fowleri*, that can be found in various terrestrial and aquatic environments. Characterizing and monitoring water bodies is crucial for determining the presence of pathogenic microorganisms and assessing the risk of infection. In this study, we propose an environmental survey to identify physicochemical parameters related to the presence of *Naegleria* in natural freshwater sources used for recreation in Costa Rica, as well as people’s knowledge, attitudes, and practices (KAP) profile in relation to the infection by *N. fowleri*.

**Materials and methods:**

Water and sediment samples were collected from 24 locations. Parameters such as temperature, pH, electrical conductivity, and dissolved oxygen in water were measured. In sediment, analyses were performed for metals, cations, and texture. Water samples were also filtered, cultured, and subjected to molecular analyses to determine the presence of FLA. Additionally, a KAP survey was conducted among 72 individuals during the fieldwork.

**Results:**

FLA were isolated at 42 °C from 22 sites, 7 of which tested positive for *Naegleria*. Although some parameters were statistically associated (*p* < 0.05) with the presence of *Naegleria*, epidemiological association was not demonstrated. From the KAP survey, 37.5% of participants had never heard about *N. fowleri*. Average scores of 35.8% for knowledge, and 3.01 and 2.16 for attitudes and practices were obtained, with 5 being the optimum value. Significant differences (*p* < 0.05) between the level of knowledge by gender and geographic origin were obtained.

**Conclusion:**

This study represents a first monitoring effort to determine the frequency of FLA in surface waters of Costa Rica, indicating the presence of thermotolerant vahlkampfiid FLA in non-thermal freshwaters with the ability to proliferate at high temperatures. The KAP survey revealed a low level of knowledge and deficiencies in the management and prevention of PAM risk among the population exposed to natural freshwater reservoirs. These findings must drive health promotion, communication, and education strategies in the local population exposed to risky recreational activities.

## 1 Introduction

Free-living amoebae (FLA) are microorganisms widely distributed in nature; some of them are considered amphizoic, since they can also cause infections (central nervous system, eyes or skin) in humans, under certain circumstances (1, 2). The main FLA with pathogenic potential belong to the genera *Acanthamoeba*, *Naegleria*, *Sappinia*, and *Balamuthia* (3). Given their ubiquity, they can inhabit aquatic environments such as rivers and lakes, which constitute a potential source of infection for people who come into contact with these sites (4, 5). One of the conditions caused by FLA is primary amoebic meningoencephalitis (PAM), an infection provoked by the thermophilic amoeba *N. fowleri*. This infection requires careful management because of its very acute course and is fatal in most cases. Furthermore, its diagnosis is not standardized, and there is no defined treatment regimen. In general, it is a poorly understood disease that is rarely suspected (5–9).

Despite the intrinsic difficulties in establishing epidemiological indicators around this event, the global occurrence has attempted to be elucidated in recent years: a total of 381 cases, 32 survivors and a fatality rate of 92% have been recorded from 1962 to 2018, according to the FLA surveillance system of the Centers for Disease Control and Prevention and consultation in bibliographic sources (2). With the same aim, in 2020, Maciver et al. (10) reported 431 cases from the literature, many of them occurring as part of outbreaks, with a 95% case fatality rate and only 21 cases successfully treated. This discrepancy also exposes the problem of unsystematic record keeping, which leads to erroneous counts and underestimations. Based on known risk factors for PAM and deaths from unspecified neurological infections, an estimate of possible underdiagnosed cases of PAM in the USA was made, with 16 deaths annually from 1999 to 2010, which fit the typical pattern of the clinical picture (11).

PAM has been reported in about 33 different countries and is most frequently found in young males (mean age of 14 years old, with a range from 1 month to 85 years old) (5). Among the main determinants associated to cases described are aquatic activities (mainly swimming and diving, recreational bathing, sports, nasal irrigation, splashing), as well as exposure to water sources such as lakes, puddles, swimming pools and, to a lesser extent, tap water, canals, ditches, rivers, hot springs and other artificial water reservoirs (5). Consequently, the risk of infection increases during recreational activities involving contact with freshwater bodies, particularly when elevated water temperatures are present. Such temperature increases may result from natural factors, such as local climatic conditions or anthropogenic sources, including thermal pollution from power plants (12–15). This indicates that monitoring water sources in which humans may come into contact to pathogenic FLA is essential, given the risk posed by the amoeba’s characteristics and the epidemiological evidence of its occurrence.

Knowledge, attitudes and practice (KAP) survey models have been widely used in public health studies since they allow the establishment of a starting line for designing health promotion policies, prevention and educational programs, as well as measuring the effectiveness of interventions, based on the findings of behavior, barriers and the social response to certain phenomena (16). In emerging events, such as the case of diseases caused by FLA, establishing a basis for social understanding allows us to obtain valuable information for preventive actions (17).

Furthermore, due to the environmental contribution to the disease burden, an environmental surveillance system is necessary to understand the distribution, abundance and frequency of *N. fowleri* and other FLA in aquatic environments. This allows to guide prioritization and intervention of decision makers. A meta-analysis of data from 35 countries yielded a global prevalence of *Naegleria* spp. and *N. fowleri* of 26.4 and 23.3%, respectively, in different water sources (18). The characterization of the elements of the environment and their role in the causal pathway to a phenomenon allows to elucidate the exposure within the framework of environmental epidemiology, as well as redirecting the focus to the understanding of the exposure (not only to the effect), giving visibility to the context of the conditions that determine the events (19).

Some researchers have proposed the development of surveys to determine and monitor the presence of *N. fowleri* in the surface waters of regions affected by cases of PAM (20, 21). Environmental condition surveys have been conducted in several human-modified water sources, such as pipes, artificial pools or wells, where potentially pathogenic FLA have been found with no findings of *N. fowleri* (22–26). Negative results have also been obtained for *N. fowleri* in natural water bodies, but with a significant presence of other *Naegleria* species (27–31). However, it has been possible to corroborate the presence of the pathogenic amoeba in rivers of important cities such as El Cairo (Egypt) (higher prevalence in summer and spring) (32) and Daejeon (Korea) (33), in ponds in India (34), as well as in lakes (35–37) and waters associated to recreation centers, such as swimming pools, hot springs, rivers and ponds in tourist complexes (38, 39). Many outstanding works of this type have included the determination of biotic and abiotic parameters to correlate with the discovery of the amoeba and provide more far-reaching information to understand the phenomenon (1, 40).

During sampling of water or sediments from rivers or lakes, measurements of temperature, pH, electrical conductivity, turbidity and dissolved oxygen concentration have been performed. Although a relationship between these measurements and the presence of *N. fowleri* has not been established in many cases (33, 36, 37) even those carried out in positive underground aquifers (38), there are studies that reflect positive correlations with temperature (15, 41). The role of some compounds in promoting amoeba development has also been proposed. For example, the addition of exogenous iron to the culture medium has favored viability and growth, whereas iron chelating agents have an inhibitory effect (42, 43). Manganese has also been studied as a possible favorable element for the growth of amoeba (44). Other elements tested as potential predictors of the presence of *N. fowleri* in recreational waters, with highly variable results and negative correlations, have been potassium, calcium, magnesium, sulfate, chloride, sodium, ammonium, nitrate, bicarbonate ions, total carbon and silicon dioxide (39).

In addition, other studies have found positive relationships between the presence of the amoeba and turbidity in recreational geothermal waters, which could be associated to the presence of suspended sediment (39). Finally, the texture of sediments, which is the relative composition of particles of different sizes in terms of sand, silt, and clay, indicates the provenance and porosity (45), but also correlates with microbial activity (46).

Given the ecological and behavioral component of the disease, the objective of this work was to identify physicochemical parameters related to the presence of FLA of the genus *Naegleria* capable of growing at high temperatures in different natural recreational freshwater sources in Costa Rica during 2023, as well as the profile of visiting people regarding knowledge, attitudes and practices concerning to *N. fowleri* infection.

## 2 Materials and methods

### 2.1 Sampling and study area

An environmental conditions survey was proposed as part of a descriptive and analytical cross-sectional study of natural freshwater bodies located in the Chorotega, Huetar Norte and Huetar Caribe regions, epidemiologically linked to PAM cases in Costa Rica (47, 48). According to Solano Quintero & Villalobos Flores (49), as in the Regional Action Plans for Climate Change Adaptation of “Ministerio de Ambiente y Energía (MINAE) (50–52), the Chorotega region has areas of temperate climate and others of dry tropical climate, with temperatures ranging between 21 °C and 36 °C, while the Huetar Norte and Huetar Caribe regions exhibit a humid tropical climate and average temperatures of 26 °C and 27 °C to 30 °C, respectively.

A minimum sampling frame of twenty sites was defined for non-probability convenience sampling, identified by consulting the Chambers of Tourism of the regions of interest. These water bodies were recognized as tourist destinations with public access, free of charge, or managed by a Community Development Association, and their topography allowed for access. All the identified sampling sites were visited, and the sample number was expanded using the snowball method (53), which involved locating nearby sites that met the selection criteria outlined above, resulting in a total of twenty-four sites. Even when sampling method limits representativeness of results, this work did not aimed inference for the whole country but to set a precedent of a FLA monitoring approach. All sampling sites were georeferenced using a mobile application with a global positioning system: TcpGPS (Aplitop - Surveying & Civil Engineering Solutions, Spain) (54). These data allowed the creation of a point shapefile for representing the freshwaters’ location in QGIS 3.30.3 (55).

Four samples of water were taken from each water body, which were later combined into a single composite sample representative of the site. Sediment sampling was carried out in the same manner. Water samples were collected according to the procedures outlined in the Standard Methods for the Examination of Water and Wastewater 2017 (56), with some adaptations from the methodology proposed by Lares Villa et al. (38), including a depth of 30 cm. The sediment was also removed and allowed to settle before sample collection in sterile 450 mL bags (LABPLAS Inc., Canada). The samples were transported and stored at room temperature until they were processed in the laboratory.

Sediment samples were collected (at 0–10 cm depth) and stored in four sterile 50 mL plastic tubes (Boeckel & Co. GmbH & Co. KG, Hamburg, Germany), according to the Sediment Sampling Guide and Methodologies 2012 (57).

### 2.2 Isolation and identification of FLA

For FLA isolation and identification, the procedure described by Retana Moreira et al. (48) was employed, with some modifications. Briefly, the total final volume of each composite water sample (1.8 L) was vacuum filtered through 0.45 μm pore size nitrocellulose membranes (Merck-KGaA, Darmstadt, Germany), and each filter was plated onto 1.5% non-nutrient agar plates supplemented with *Escherichia coli*. The plates were incubated at 42 °C for at least 7 days, and growing amoebae were subcultured by transferring a small amount of the culture media to fresh medium for selection and propagation at 42 °C for 2–3 days. Then, the growing plates were washed with 1.0 mL of cold, sterile phosphate-buffered saline solution, and the washed material was transferred to 1.5 mL tubes and centrifuged at 3000 × g for 10 min. The supernatant obtained was discarded, while the pellet was employed for molecular testing.

DNA extraction was performed using the pellet obtained from the last step with the QIAamp DNA Mini Kit (Qiagen, Hilden, Germany), following the manufacturer’s instructions. To ensure quality of DNA samples, the products were quantified using a NanodropTM 2000 spectrophotometer (Thermo Fisher Scientific, MA, USA) and stored at −20 °C until use.

The complete ITS region (ITS1, ITS2, and 5.8S) was amplified according to De Jonckheere & Brown (58). For this purpose, specific primers for Vahlkampfiidae, the taxonomic family of *N. fowleri* (Vahl-F: 5’-GTCTTCGTAGGTGAACCTGC-3′, Vahl-R: 5’-CCGCT TACTGATATGCTTAA-3′), and for genus *Naegleria* (ITSFW: 5´-AACCTGCGTAGGGATCATTT-3′, and ITSRV: 5’-TTTCCTCC CCTTATTAATAT-3′) were employed. PCR and electrophoresis were also performed as described by Retana Moreira et al. (25). Negative controls (distilled water) and positive controls (DNA from the *N. fowleri* Lee ATCC 30808 strain) were used.

### 2.3 Measurement of physicochemical parameters

Physicochemical parameters including temperature, dissolved solutes, pH and electrical conductivity were measured at each sampling site using a YIERYI C-600 multiparameter meter (Shen Zhen Yage Technology Co., China). Dissolved oxygen concentration was also determined using a YIERYI JPB-70A analyzer (Shen Zhen Yage Technology Co., China). Chemical analyses (calcium, magnesium, potassium, phosphorus, copper, iron, zinc, manganese, carbon, and nitrogen), pH, electrical conductivity and texture of the sediment were performed at Centro de Investigaciones Agronómicas from the Universidad de Costa Rica (UCR). All determinations were performed in triplicate, except for sediment texture analyses.

### 2.4 KAP exploratory survey on *N. fowleri* infection and its risk

At the time of visit of each sampling site, tourists of legal age (18 years and older) were selected to administer a questionnaire. Prior to administering the questionnaire, participants were provided with information about the study to obtain their consent to participate. All data was collected anonymously. For this purpose, a structured questionnaire was designed, featuring closed and pre-coded questions, through which information was collected to build the demographic and KAP profiles of people visiting each sampled water body. Questions included information about the clinical picture of PAM, knowledge about the risk and measures to prevent an infection caused by *N. fowleri*. The work of Shakeel et al. (59) is closely related to the object of study in this work, allowing us to establish the foundation for this tool. However, in other topics related to communicable diseases, there are also validated surveys (60) that contribute to areas such as the one discussed here. A sociology expert reviewed the instrument to assess and correct the type and quality of the questions, as well as the clarity of the concepts.

Knowledge about amoeba, PAM, and ways to prevent the infection were assessed by the number of correct answers on the topic (61). Behavior, in terms of attitudes and practices toward the infection and the risks of acquiring it in recreational settings, was measured using a Likert scale, where a series of statements and possible responses were posed: “totally agree/always,” “agree/most of the time yes,” “neither agree nor disagree/sometimes yes, sometimes no,” “disagree/most of the time no,” and “totally disagree/never,” which were scored from five to one, respectively (62). Risk behavior was scored by dividing the total score by the number of statements (63).

### 2.5 Statistical analysis

Descriptive statistics were employed to summarize, organize and present the data. The Shapiro–Wilk normality test was used to assess the distribution of quantitative variables and allowed the definition of bivariate inferential statistics.

For environmental variables, the dependent variable was *Naegleria* positivity at the sampled sites. In contrast, the independent variables were the results of physicochemical determinations in water and sediment. Parametric (Student’s t-test, *α* = 0.05) and non-parametric (Wilcoxon Rank Sum test, α = 0.05) tests were applied to compare measures of central tendency between groups defined by the dependent variable. To establish a model of explanatory relationships for the dependent variable, a multivariate, non-conditional logistic regression was employed, from which odds ratios (OR) and their respective 95% confidence intervals (95% CI) were obtained. The candidate variables to be integrated (including data from sediment and water) into the model using the stepwise method were selected based on scientific evidence and the Hosmer-Lemeshow criterion (*p* < 0.25) (64) following bivariate analysis. The best-fitting model was selected according to the Akaike Information Criterion (65).

To analyze the survey data, sociodemographic information was classified by gender (female, FE; male, MA) and age group (variable recategorized into two groups based on the numerical data of age, focused on the groups proposed by Martín Ruiz (66): 18–39 years and ≥ 40 years, separating young adults from middle/mature adults). Categories of geographic origin were also generated based on non-resident foreign status declared during the survey, and in accordance to the update of the Regional Plan for the Great Metropolitan Area (GAM) 2013–2030 (N° 38,334-PLAN-MINAE-MIVAH-MOPT-S-MAG) (67) to classify residents as being from the GAM or not. Non-parametric statistics were also used to test the association between the KAP variables (Spearman’s Rho test, *α* = 0.05) and other categorical variables such as age group, gender (Wilcoxon Rank Sum Test, α = 0.05), and geographic origin (Kruskal-Wallis test, *α* = 0.05). For the knowledge attribute, the correct answer frequencies were compared to the categories of the variables: geographic origin, gender and age group (Pearson’s Chi-square test, *α* = 0.05).

All analyses were performed using the statistical package STATA® v. 14 (Stata Corp LLC, TX, USA) (68).

## 3 Results

### 3.1 Isolation and identification of FLA

Twenty-four water bodies were sampled between July and November, 2023. Ninety-six water samples were collected, of which 56 (from 22 sampling sites) showed positive growth of FLA at 42 °C. The different morphotypes from each site were selected and subcultured for identification by molecular methods.

**Figure 1:**
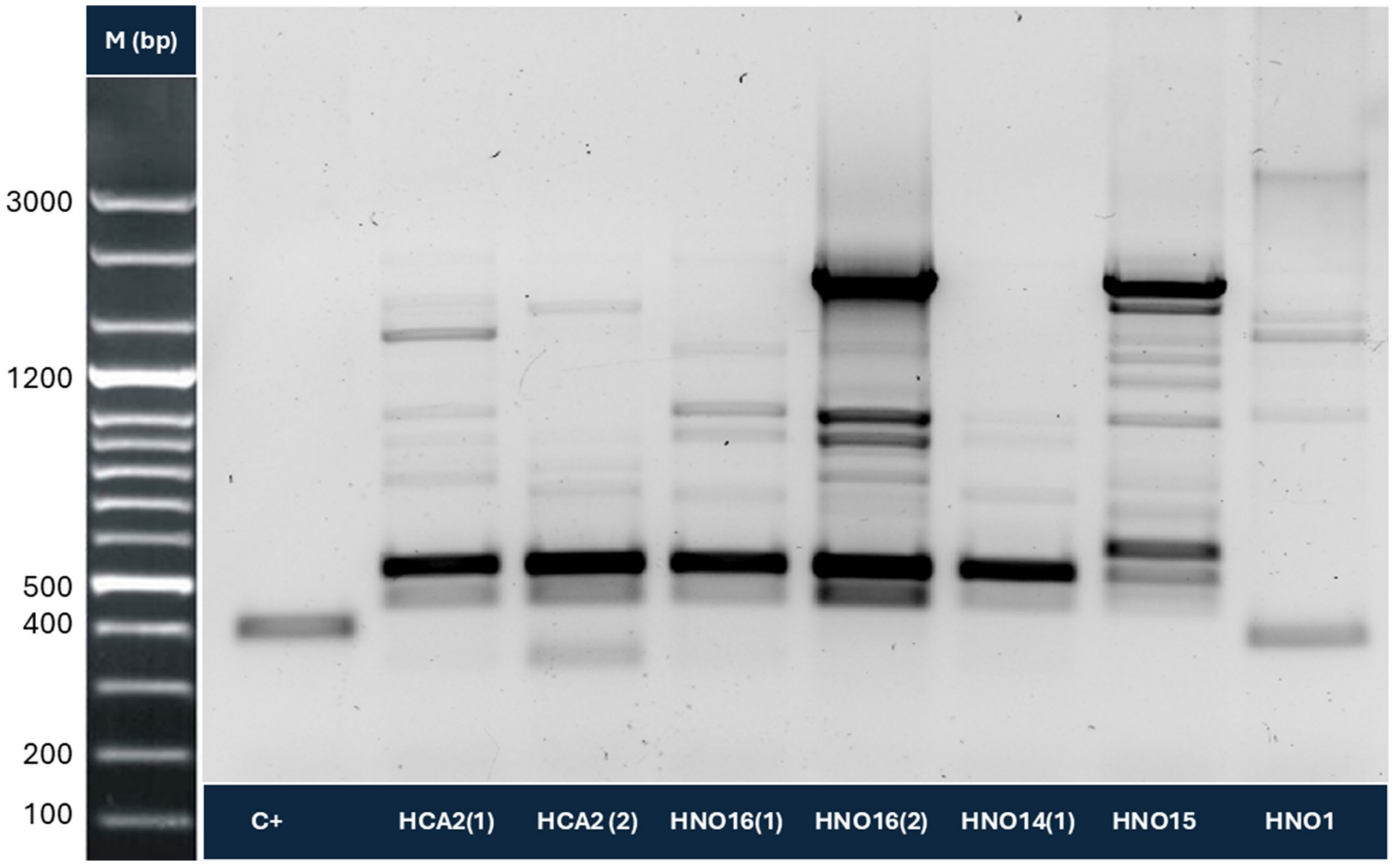
Molecular identification of *Naegleria* (ITS region) in water samples. In this Figure, seven of the eleven positive samples are shown, representing two of the three regions included in this study. M: molecular weight marker in base pairs (bp); C+: positive control (*N. fowleri* DNA).

**Figure 2:**
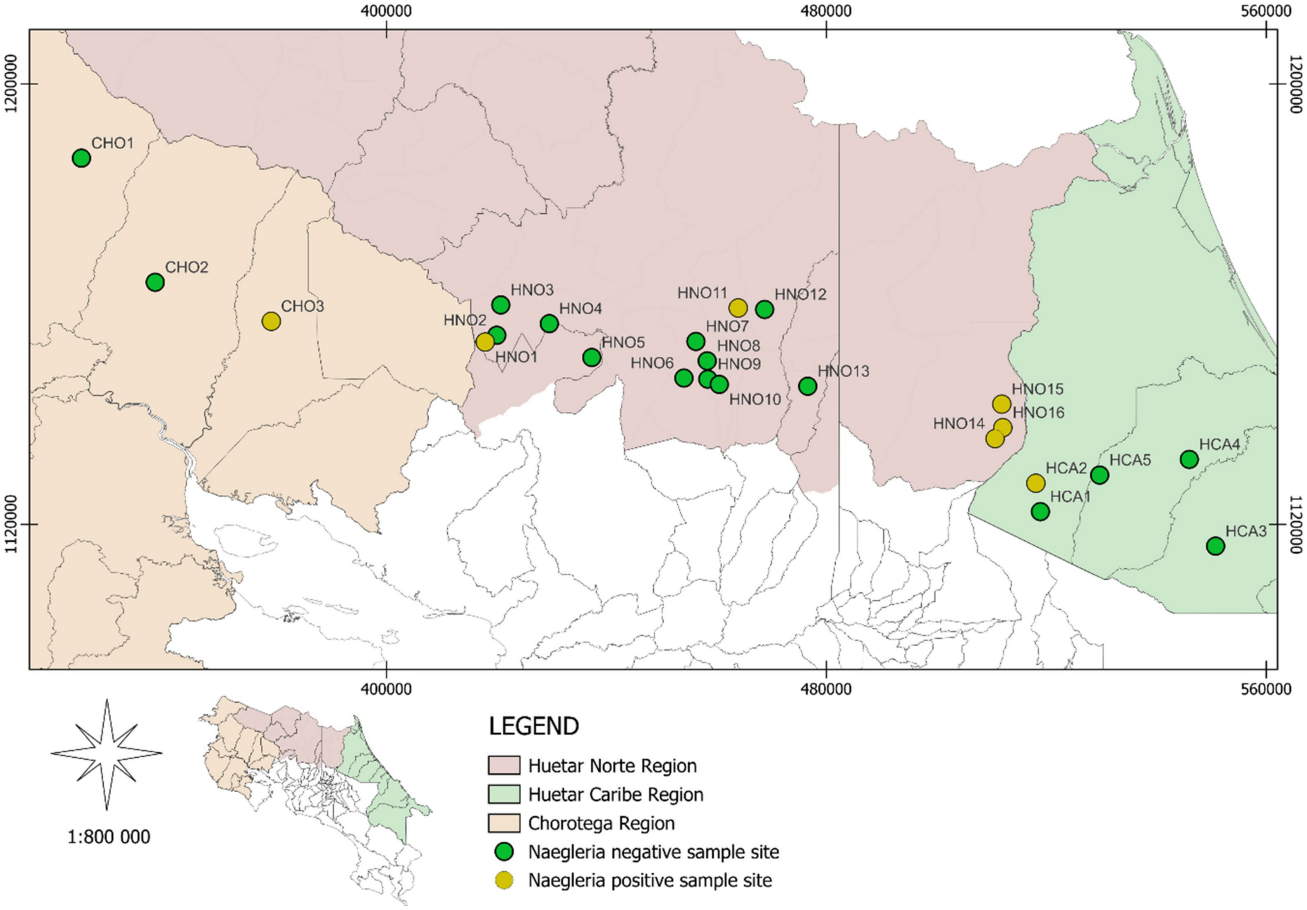
Sampling sites where FLA were isolated and identified as *Naegleria* by PCR. Of the water samples taken at all these sites, only HCA1 and HNO2 were negative for FLA growing at 42 °C. The regions that constituted the study areas are indicated.

PCR for the Vahlkampfiidae family was positive in isolates obtained from 20 sampling sites (Table 1). PCR at the *Naegleria* genus level revealed 11 positive subsamples from seven sampling sites (five from the Huetar Norte Region, one from the Chorotega Region and one from the Huetar Caribe Region), as shown in Figures 1, 2, for a relative frequency of 29.2% (7/24) (95% CI: 11.0–47.4). For some of these positive *Naegleria* samples, a band close to that of the *N. fowleri* positive control was obtained (around 400 bp).

**Table 1:** PCR detection of Vahlkampfiidae (VAHL) and *Naegleria* in recreational freshwaters of three regions of Costa Rica.

Site codification	Percentage (%) of samples for each site (*n* = 4) with FLA growth at 42 °C	VAHL-PCR	*Naegleria*-PCR
Chorotega region			
CHO1	50	POS	NEG
CHO2	50	POS	NEG
CHO3	50	POS	POS
Huetar Caribe region			
HCA1	0	NA	NA
HCA2	25	POS	POS
HCA3	100	POS	NEG
HCA4	100	NEG	NEG
HCA5	100	POS	NEG
Huetar Norte region			
HNO1	50	POS	POS
HNO2	0	NA	NA
HNO3	75	POS	NEG
HNO4	100	POS	NEG
HNO5	100	POS	NEG
HNO6	50	NEG	NEG
HNO7	100	POS	NEG
HNO8	75	POS	NEG
HNO9	75	POS	NEG
HNO10	25	POS	NEG
HNO11	75	POS	POS
HNO12	25	POS	NEG
HNO13	50	POS	NEG
HNO14	50	POS	POS
HNO15	25	POS	POS
HNO16	50	POS	POS
Relative frequency (*n* = 24)	91.7%	83.3%	29.2%

POS: positive, NEG: negative, NA: not applicable.

An analysis based on the positivity of the sites for *Naegleria* (taxonomic genus) was proposed as an approximation of the presence of potentially pathogenic FLA.

### 3.2 Physicochemical characterization of water and sediments from freshwater bodies

Water and sediment measurements from each water body are detailed in Table 2. Sediment classification by textural class is specified in Table 3. From these results, sandy was the most frequent type of texture found. Regarding physicochemical parameters, only the dissolved oxygen and phosphorus measurement data sets followed a normal distribution, as indicated by the Shapiro–Wilk test (*p* > 0.05). No statistically significant associations were found between quantitative variables and the presence of *Naegleria* (*p* > 0.05), except for electrical conductivity and dissolved oxygen in water, as well as copper determination and the percentage of clay in the sediment.

**Table 2:** Physicochemical parameters measured in water and sediment, according to their molecular positivity for *Naegleria*.

Physicochemical parameter	Measure unit	POS (*n* = 7)		NEG (*n* = 17)		Total (*n* = 24)	
		Mean (SD)	Median (IQR)	Mean (SD)	Median (IQR)	Mean (SD)	Median (IQR)
Matrix: water							
Temperature	°C	25.0 (2.0)	25.4 (3.4)	26.0 (3.6)	24.1 (4.1)	25.7 (3.3)	25.0 (3.8)
Dissolved solutes	ppm	34.3 (12.3)	28.0 (25.0)	121.1 (198.3)	51.0 (74.3)	95.8 (170.4)	44.2 (26.8)
Electrical conductivity **	μS/cm	69.0 (24.1)	56.0 (49.3)	233.7 (372.3)	102.7 (149.3)	185.6 (320.0)	93.3 (54.4)
Dissolved oxygen *	mg/dl	12.5 (1.4)	13.1 (2.4)	11.3 (1.2)	11.4 (1.3)	11.7 (1.4)	11.7 (1.8)
pH		7.1 (0.4)	7.0 (0.9)	6.8 (0.3)	6.7 (0.2)	6.8 (0.4)	6.7 (0.4)
Matrix: sediment							
pH		6.8 (0.4)	6.7 (0.2)	6.8 (0.9)	7.0 (0.5)	6.8 (0.8)	6.8 (0.6)
Ca	cmol(+)/l	5.5 (2.7)	4.5 (3.1)	5.8 (3.8)	5.3 (2.1)	5.7 (3.4)	5.3 (2.1)
Mg		2.3 (1.0)	2.1 (0.9)	2.8 (2.9)	1.8 (1.5)	2.7 (2.5)	2.0 (1.4)
K		0.2 (0.1)	0.2 (0.1)	0.4 (0.2)	0.3 (0.3)	0.4 (0.2)	0.3 (0.2)
P	mg/l	3.3 (1.1)	3.0 (2.0)	3.7 (1.7)	3.0 (3.0)	3.6 (1.5)	3.0 (3.0)
Zn		1.1 (1.0)	1.0 (1.6)	2.1 (1.7)	1.9 (2.3)	1.8 (1.6)	1.2 (2.4)
Cu **		1.4 (1.3)	1.0 (3.0)	3.6 (3.1)	3.0 (1.6)	2.9 (2.9)	2.5 (2.0)
Fe		29.1 (17.4)	21.0 (33.0)	51.7 (62.4)	32.0 (49.0)	45.6 (53.8)	32.0 (38.8)
Mn		6.4 (4.9)	5.0 (10.0)	14.2 (26.5)	6.0 (9.0)	11.9 (22.5)	6.0 (8.2)
Electrical conductivity	mS/cm	0.1 (0.0)	0.10 (0.0)	0.2 (0.1)	0.1 (0.1)	0.1 (0.1)	0.1 (0.0)
Sand	%	93.7 (2.4)	95.0 (3.0)	87.1 (12.4)	91.0 (4.9)	89.0 (10.9)	92.0 (5.0)
Silt		4.7 (1.2)	5.0 (3.0)	6.7 (5.7)	4.0 (5.0)	6.1 (4.9)	5.0 (3.8)
Clay **		1.6 (1.8)	2.0 (2.0)	6.2 (8.0)	5.0 (4.0)	4.8 (7.1)	2.0 (3.8)

SD: standard deviation, IQR: interquartil range.

* T-test (*p* < 0.05), the difference in means is statistically significant. ** Wilcoxon Rank-Sum Test (*p* < 0.05), the difference in medians is statistically significant.

**Table 3:** Classification of sampled sediments according to texture types and the site molecular positivity for *Naegleria*.

Texture class	POS (*n* = 7)	NEG (*n* = 17)	Total (*n* = 24)
Sandy	7	13	20
Loamy sand	0	2	2
Sandy loam	0	1	1
Clay loam	0	1	1

### 3.3 Multivariate logistic regression model

With the variables included under the characteristics of this study, an epidemiological model with the appropriate statistical fit was not achieved to define factors that can be interpreted, using OR, as having a greater or lesser influence on the likelihood of *Naegleria* presence in freshwater bodies (Supplementary Table S1). In this sense, the epidemiological association measures did not show statistical significance.

### 3.4 KAP exploratory survey on *N. fowleri* infection and its risk

Surveys were administered at 20 of the 24 sampling sites to a total of 72 individuals: 45 women and 27 men, aged 18–62 and 18–66 years old, respectively. The distribution by gender, age group and geographic origin is shown in Table 4. Results revealed that 56.9% (41; 95% CI: 45.5–68.4) did not specify a preferred month for visiting the sites for recreational purposes; however, March and April were the most frequently mentioned as specific months. Also, 77.8% (56; 95% CI: 68.2–87.4) reported regularly visiting these types of sites for recreation at least once a year, and 61.1% (44; 95% CI: 49.9–72.4) reported being non-local visitors to the community where the freshwater body is located.

**Table 4:** Frequencies of sociodemographic aspects of recreational freshwater visitors surveyed during sampling at 20 collection sites.

Sociodemographic profile component	Have heard of *Naegleria fowleri* or its vernacular names		Total number of people surveyed (*n* = 72)
	Yes	No	
	45 (62.5%)	27 (37.5%)	
Gender (absolute frequency)			
Female	32	13	45
Male	13	14	27
Gender (relative frequency, %)			
Female	44.5	18.0	62.5
Male	18.0	19.5	37.5
Age group (absolute frequency)			
18–39 years old	30	15	45
≥ 40 years old	15	12	27
Age group (relative frequency, %)			
18–39	41.7	20.8	62.5
≥ 40	20.8	16.7	37.5
Geographic origin (absolute frequency)			
GAM	22	9	31
No GAM	21	16	37
Foreign	2	2	4
Geographic origin (relative frequency, %)			
GAM	30.5	12.5	43.0
No GAM	29.2	22.2	51.4
Foreign	2.8	2.8	5.6

GAM: Great metropolitan area, No GAM: outskirts of metropolitan area.

**Figure 3:**
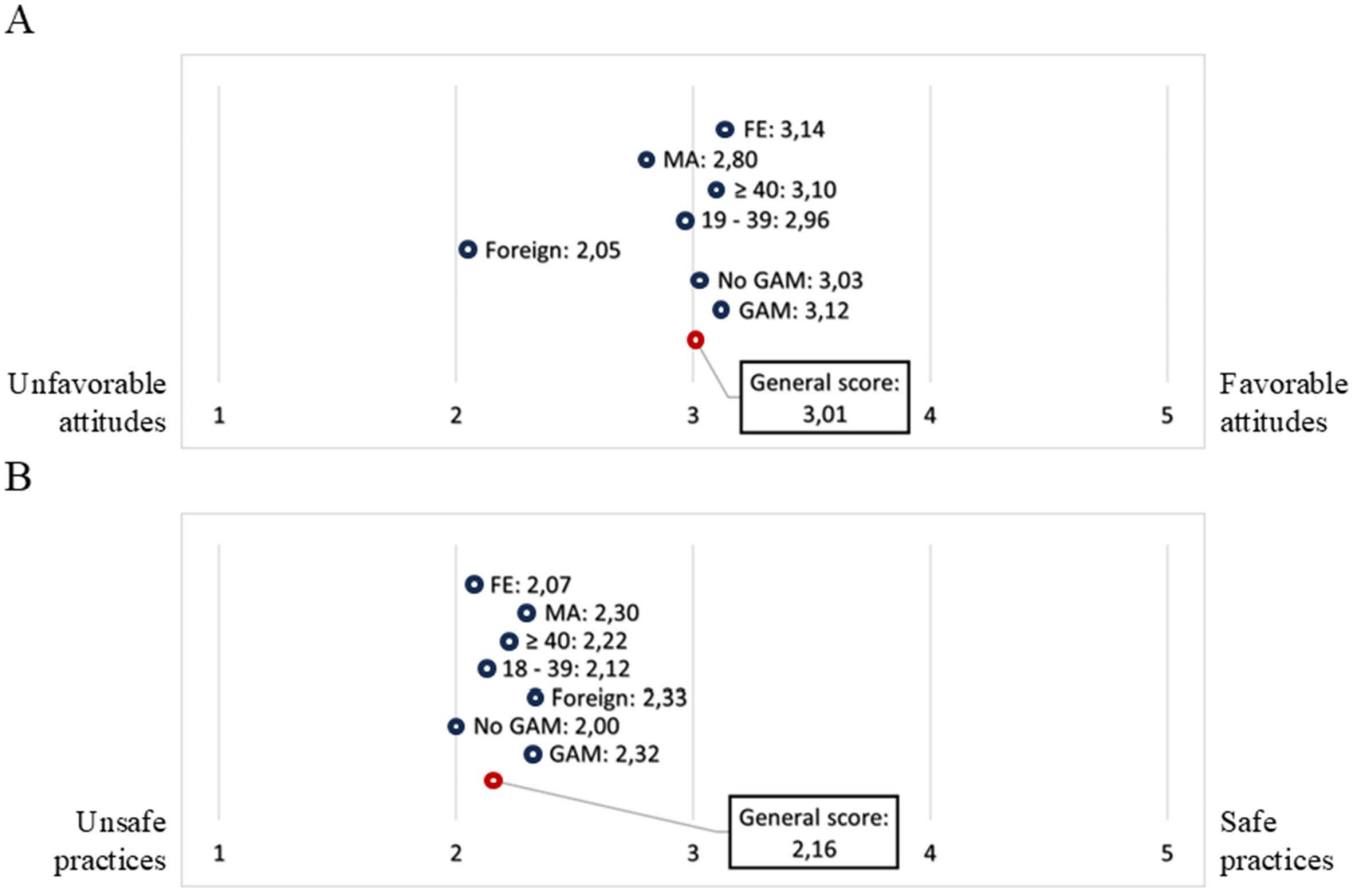
Likert scale for assessing behavior related to the risk of *Naegleria fowleri* infection in recreational freshwater sites. The overall score obtained for attitudes **(A)** and practices **(B)** is included, as well as the score for each component after disaggregation by category of geographic origin (GAM: Great Metropolitan Area, No GAM: outskirts of metropolitan area, foreign), age group (18–39 years old: young adults, ≥ 40 years old: middle and mature adults) and gender (MA: male, FE: female).

Regarding knowledge, it is noteworthy that 62.5% (95% CI: 51.3–73.7) of respondents had heard of *Naegleria fowleri*, also known as the “brain-eating amoeba,” while 37.5% (95% CI: 26.3–48.7) reported that they had not. The percentage of correct responses (knowledge score) by item is shown in Table 5. The Likert scaling for attitudes and practices assessment by gender, age group and geographic origin is shown in Figure 3. The absolute number of response types obtained per item is shown consolidated in Supplementary Table S2. From these results, a medium positive correlation was observed (Spearman’s rho test = 0.3571; *p* = 0.002) between the knowledge score and the attitude score. No statistically significant correlations were found between the numerical variables A-P or C-P, nor were there any associations with the categorical variables, except for the comparison of the median knowledge scores between genders and geographic origin (*p* < 0.05).

**Table 5:** Knowledge assessment of *Naegleria fowleri* infection in people visiting freshwater bodies for recreation.

Specific knowledge that the item evaluates	Score	Disaggregation by						
		Gender *		Age group		Geographic origin *		
		FE	MA	18–39	≥ 40	GAM	No GAM	Foreign
Route of entry into the body for acquiring the infection	30.6	35.5	22.2	35.5	22.2	38.7	24.3	25.0
Main clinical manifestations of the infection	36.1	44.4	22.2	35.5	37.0	54.8	24.3	0.0
Frequency of cases in the country	37.5	44.4	25.9	37.8	37.0	51.6	27.0	25.0
Severity of the infection in humans	44.4	57.8	22.2	46.7	40.7	58.1	35.1	25.0
Places where the infection is likely to be acquired	30.6	33.3	25.9	33.3	25.9	35.5	27.0	25.0
Overall/general score	35.8	43.1	23.7	37.8	32.6	47.7	27.6	20.0
*p* value		< 0.001*		0.321		< 0.001*		

* *χ*^2^ (*p* < 0.05), test of dependence between the correct answers and the categorical variable is statistically significant.

## 4 Discussion

### 4.1 Presence of *Naegleria* in recreational freshwaters and its environmental determinants

FLA are ubiquitous microorganisms, present in a wide variety of aquatic and terrestrial environments (1, 2). In this study, FLA capable of growing at 42 °C were isolated from water samples from 22 sites distributed throughout the Chorotega, Huetar Norte and Huetar Caribe regions of Costa Rica. Furthermore, conventional PCR determined that seven of these sites were positive for *Naegleria*.

The overall proportion of sites positive for *Naegleria* in this study was 29.2% (7/24) (95% CI: 11.0–47.4), which is close to the results of environmental conditions surveys where work has been carried out with more than 100 water bodies (34); moreover, prevalences of 23.1% of FLA, 34.6% of *Naegleria* spp. and 0.9% of *N. fowleri* have been found (34). In another similar study, 23.1% of *Naegleria* spp. and 1.9% of *N. fowleri* were reported (69). Specifically, for *N. fowleri* in the Americas, there are reports of prevalences from 2.0% (95% CI: 0.2–7.0) (Brazil), 23.9% (95% CI: 10.0–40.8) (Mexico) and up to 24.1% (95% CI: 15.0–34.4) (USA) (70). There are also global prevalence data from sites like those included in this work, such as rivers with 19.7% (95% CI: 10.3–30.9%) and lakes with 33.0% (95% CI: 12.6–56.7%) of positivity (70).

Regarding the determination of physicochemical parameters traditionally associated to the presence of *N. fowleri*, temperature oscillated around 25 °C, a result that is noteworthy since the majority of samples were collected from natural, non-thermal water bodies. According to the literature, although temperatures above 30 °C are associated with a greater number of isolations of this amoeba, it has also been reported that cystic forms can be found in the bottom sediments of water bodies at temperatures as low as 12 °C (71). Isolation has also been possible during periods of falling atmospheric temperature with counts as high as 201 NMP/l (37, 38).

In a study carried out in the Monjolinho River, Brazil, thermotolerant amoebas such as *N. australiensis* and *N. philippinensis* were isolated and identified from waters with temperatures between 17.8 °C and 20.9 °C (28); in Mexico, *N. fowleri* and *N. australiensis* were found in irrigation canals at temperatures of 17 °C (72). For this reason, it is crucial to monitor waters over a wide range of temperatures and to consider the effect of other covariates that may influence the occurrence of the amoeba (40). Coincidentally, in this case, the *Naegleria*-positive sites had temperatures below 28 °C. Another remarkably stable water parameter was the pH, with values around 6.8, which falls within the range of previous findings or *in vitro* studies using *N. fowleri* (40, 69, 73, 74).

Regarding measurements in the sediments at the sampling sites, the data showed high variability, as indicated by the dispersion measures, particularly in the determinations of cations, metals, silt and clay. However, and in accordance to the literature reviewed, it is essential to highlight that this is the first attempt to correlate sediment parameters to the presence of *Naegleria* in water associated to this sediment, based on the hypothesis that these amoebae persist in these matrices rich in bacterial mats such as biofilms, which constitute a food source (40, 75).

The importance of metals such as iron for *N. fowleri* has already been proven (76). Iron seem to favor the presence of this amoeba, since it has been found in layers of lakes rich in the metal, but it may also contribute to its pathogenicity (77). Besides, Mach et al. (78) demonstrated that, under conditions of low iron availability, *N. gruberi*, a non-pathogenic amoeba related to *N. fowleri*, responds with compensatory metabolic mechanisms. In this study, bioavailable iron concentrations in sediments were the highest compared to the other determinations at all sampling sites, so this variable may not have made a difference in the distribution of *Naegleria*.

Since other species of the genus *Naegleria* share characteristics of survival at high temperatures and/or pathogenicity, such as *N. lovaniensis*, *N. australiensis* and *N. italica* (79, 80) and, as an approximation of the possibility of the presence of *N. fowleri* in the analysed water bodies, bivariate inferential statistical analyses were performed between the multiple environmental data collected (as independent variables) and *Naegleria* positivity, resulting in statistically significant associations with electrical conductivity and dissolved oxygen determinations in water. These results describe a higher concentration of dissolved oxygen in *Naegleria*-positive sites, which contrasts with some literature that describes negative correlations between *N. fowleri* abundance and dissolved oxygen. Since this parameter is affected by water temperature, it should be interpreted with caution (40).

Under conditions like those presented in our study, with a majority of temperatures between 20 °C and 30 °C, other authors have obtained positive correlations to dissolved oxygen in water (35, 81), but not with electrical conductivity. Increases in electrical conductivity can be associated to the presence of organic matter and anthropogenic activities that raise the concentration of compounds such as potassium, magnesium, calcium, carbonates and sulfates, so it can be employed as an indicator of pollution (Baird C, Cann M. Environmental Chemistry 5th Ed., 2012; as cited in: Bellini et al. (28)) (82). However, findings correlating the presence of *N. fowleri* have been variable (40). In this case, the parameter was significantly lower in *Naegleria*-positive sites, so it may be considered a benefit due to improved water quality.

There was also statistical significance in comparisons to copper concentration and percentage of clay in the sediment (both parameters were lower in the presence of *Naegleria*). Although copper is recognized as a crucial element for biological processes in *Naegleria* spp., high amounts have toxic effects; furthermore, when copper is deprived, these FLA undergo metabolic adaptations (83, 84). On the other hand, 83.3% of the analyzed sediments have a sandy texture, presenting very low amounts of silt and clay. *Naegleria* positivity was only detected in locations with sediments with this sandy particulate pattern. According to Rodríguez-Zaragoza (85), texture controls the distribution of FLA, as these, in turn, affect bacterial mineralization rates and require substrates to adhere and perform this function. Results of a lower percentage of clay in sediments and the presence of *Naegleria* spp. coincide with previous results (46), which indicate greater microbial activity (bacteria and protists) in surface sediments rich in sand and low in clay. In a more recent work (86), greater bacterial abundance and diversity were observed in clay soils irrigated with water from a treatment plant. Interestingly, in this same study, the frequency of FLA, such as *Acanthamoeba* and *B. mandrillaris*, was much higher in soils with a higher proportion of sand and silt than in clay soils.

Despite the associations found when comparing measures of central tendency between groups, the epidemiological association measures using OR were not significant. It should be noted that, while the number of variables is significant, the number of records (*n* = 24) limits the association and statistical power, since when dividing the data into categories of the dependent variable, the frequency of qualitative independent variables may be very low, or the dispersion of the data set may more influence the quantitative variables. Thus, for further investigations, larger datasets, recategorization and automatic selection of variables (machine learning), and overfitting avoidance techniques should be considered in order to achieve more suitable predictions. This is consistent with previous works that have not demonstrated the predictive potential of different compounds or water quality parameters on the persistence of *N. fowleri* in natural environments (39, 87, 88).

Beyond the presence of thermotolerant FLA and the risk they represent as direct etiological agents, these organisms are considered true reservoirs for bacteria and fungi, including pathogens such as *Cryptococcus neoformans*, *Legionella* spp., *Chlamydophila pneumoniae*, *Mycobacterium* spp., *Listeria monocytogenes*, *Pseudomonas aeruginosa* and *Francisella tularensis* and emerging pathogens, such as *Parachlamydia acanthamoebae* (89). From the One Health approach, which recognizes the interconnection between human, animal and environmental health, these amoeba-microbe interactions reveal an important aspect where known and emerging pathogens can strengthen and spread by ubiquitous carriers. For these amoeba-resistant organisms, this symbiotic relationship not only provides protection from environmental conditions but also seems to improve their survival by facilitating their dissemination in diverse habitats; this clearly increases the possibility of transmission (90). Furthermore, various studies have shown that the interaction between bacteria and amoebae significantly impacts bacterial characteristics related to virulence (91) and antibiotic resistance (92), among others. From the amoeba perspective, this symbiotic relationship has been related with increased pathogenicity (cytopathic effect) in *in vitro* models (93); it has recently been reported that FLA harboring endosimbiont bacteria produce more severe keratitis (94).

Water and aquatic environments might represent a double trouble in this regard; for example, an isolation from tap water related to an *Acanthamoeba* keratitis case contained *Pseudomonas aeruginosa* (95). The microbiome diversity and abundance of amoebae clinical isolates could be driven by water as a multihabitat reservoir of microorganisms (96). Recognizing the occurrence of these types of interactions and their consequences calls for strengthening surveillance and monitoring ecosystems where these interactions can occur (drinking water networks, untreated water, recreational sites, wastewater treatment plants, among others); this would allow the establishment of preventive measures that anticipate the risk of infections associated with these organisms.

### 4.2 Human behavior component of PAM risk

Regarding the approach to the presence of *N. fowleri* at the studied sites, it is worth highlighting the finding of *Naegleria* by PCR in thermotolerant FLA (isolated and replicated at 42 °C). In this sense, as human actions and conducts are determined by their awareness, these results are complemented with the implementation of a tool to describe the level of knowledge and behavioral characteristics of recreational visitors to natural water reservoirs, about the risk of *N. fowleri* infection.

A KAP survey was applied to 72 persons during water and sediment sampling, with a higher proportion of individuals interviewed as follows: 62.5% were women (vs. 37.5% men), 62.5% aged between 18 and 39 years old (vs. 37.5% of people aged 40 years old or older), 61.1% reported not residing in the locality where the body of water is located (vs. 38.9% of locals), with regular visitation at 77.8% (vs. 22.2% of first-time visitors) and 56.9% with no particular preference for the time of year in which they recreate in this type of water reservoirs.

The difference between people from the GAM (43.1%) and from outside the GAM (51.4%) was smaller, but significant, compared to non-resident foreigners in Costa Rica who were tourists at the sampled sites (5.6%). There is limited background information on surveys of this type associated with FLA infections. Studies have been published on contact lens users, addressing some questions related to the risk of amoebic keratitis, which confirm a good level of knowledge about preventive hygiene practices (17, 61). Regarding PAM, there is a publication on a knowledge interview applied to health sciences students (59) and another KAP-type interview applied to health personnel (97), both performed outside of Costa Rica.

In this case, the target population was different, including recreational visitors to freshwater bodies. According to the literature reviewed, the only study similar to ours was a KAP survey conducted among the general population of Karachi, Pakistan (98), a place where PAM has been considered an event of great concern since 2008, as varying numbers of deaths have been reported each year since then (99). In the study presented here, only 37.5% of participants reported never having heard of *Naegleria fowleri*, also known as the “brain-eating amoeba,” indicating a lack of awareness among the population. It has been observed that for other neglected parasitic infections, awareness of the infection, its biology, prevention, and control measures is insufficient (100). This aligns with the idea that life experiences can contribute to a better sense of self-care and prevention; however, PAM is a disease that, in addition to being highly lethal, is rare in the country, and having experienced it firsthand is difficult in the Costa Rican context.

The knowledge score, operationalized as the percentage of correct answers, shows an inadequate level of information management related to the topic of interest, as none of the theoretical items exceeds 45.0%, and the overall score is 35.8%. Additionally, when analyzing the disaggregation by gender and geographic origin, significant differences were found, with higher knowledge scores for women and people from the GAM. Reinforcing the idea, a KAP study on schistosomiasis found that individuals who have suffered from or know someone who has suffered from the disease, or have participated in related health interventions, have higher KAP scores (101).

Regarding the differences between men and women, it should be noted that women have had historically more comprehensive health care; this can be explained by higher levels of health literacy resulting from: (i) better exploration of the health system and, (ii) traditional gender roles regarding family care based on expectations that have required them to have a better knowledge base (102). In agreement to the National Academies of Sciences, Engineering, and Medicine (103), women use health services more than men, but they also make more primary care visits, receive more diagnoses, screenings and nutritional and sexual health support. However, men’s risk perception can modulate their propensity to seek information, according to Manierre (104).

On the other hand, the knowledge variable was significantly lower in people from outside the GAM, which is associated with predominantly rural areas of the country. Although it has been suggested that the urban–rural disparity effect may be increased in developing countries due to the availability of health resources and is not entirely due to rurality per se (105), the urban–rural dichotomy disadvantages rural areas with less access to health services, sources of health information and fewer options for health providers, being, in turn, determined by characteristics such as the economic income and education of these populations (103, 106). It must be considered that the skills that a population has to access and assimilate health education do not necessarily translate into obtaining sufficient knowledge about a specific disease and this gap is related to access to specialized information, specialists in the area and personal experiences (105), which could occur in the case of PAM because it is a relatively emerging phenomenon.

Regarding the attitudes and practices scores, the results show unfavorable attitudes and risky practices with medium to high levels (3.01/5.00 and 2.16/5.00, respectively). Although there were no differences by gender or age group in practices or attitudes, putting into perspective that the majority of participants were between 18 and 39 years old, it should be considered that the demographic group that has been proposed as most exposed to PAM by epidemiology has been young men (5, 107, 108), These individuals are presumed to be more likely to engage in activities that facilitate the forced entry of water through the nose, such as jumping, diving or splashing, which enable the invasion of the amoeba. They are also associated with spending more time in the water, engaging in activities that stir up sediment that may contain *N. fowleri* (108, 109). In this sense, the use of nose protectors and keeping the head out of the water are well-established and widely recognized preventive measures (110).

According to these results, the use of a nose clip during water activities is the least implemented practice, and there was greater variability in responses regarding the practice of diving or submerging during water activities. One component that could explain the practices score is the fact that people seem to associate the amoeba more with hot springs. There is extensive evidence of cases related to water sources of various origins and natures, including lakes, rivers, spas, canals, and puddles, among others (107, 108, 111).

The attitudes with the variety of responses were those concerned about getting sick and attending health centers when experiencing symptoms related to the infection. It was expected that people would develop better practices and attitudes as their knowledge and understanding of PAM increased. However, knowledge and practices do not always correlate, depending on the context, as they may be mediated by determinants that limit appropriate action, for example, when there is no access to resources to implement preventive or health promotion practices (112, 113).

The only mild correlation found in this study was between knowledge and attitude, which can be associated with beliefs shaped by an understanding of the phenomenon. This result is not surprising given that the majority of people are aware of the existence of *N. fowleri*, maybe as a result of the communications issued by the Costa Rican Ministry of Health in 2020, when preventive actions were suggested, such as banning trampolines, cleaning pools, and posting signs to prevent and warn of the possible presence of *N. fowleri* (114–116). The context of these communications was focused on hot springs recreation centers; therefore, it is considered that the message biased the population and made other potential sources invisible (such as those included in this study, where the presence of other thermotolerant FLA was demonstrated), and this may influence the findings regarding practices and attitudes. Yoder et al. (108) pointed out that warning signs in certain places can create confusion and suggested that the lack of signage in other bodies of water means they are free of the pathogen.

The conducted KAP survey identified population vulnerabilities related to poor knowledge and risky behaviors that increase the risk of acquiring *N. fowleri* infection. These findings are consistent with a previous work published by Younus et al. (98), who reported 80.0% of people had never heard of the pathogen, more than 95.0% of incorrect answers when addressing knowledge and almost 100.0% who do not avoid water activities during the summer or do not prevent water from entering the nose in any way. Giving that these findings arise from a context with 29.2% of *Naegleria* and 83.3% of thermotolerant FLA presence, gaps highlighted by the KAP survey imply a misinformation bias and, then, a potential increased risk of infection in settings that meet the conditions to host possible pathogens. Together, these two approaches allow the description of these water reservoirs as nosogenic territories.

The results of this study face limitations in terms of representativeness due to the small sample size and the non-probabilistic selection of sampling units. The sampling framework for this type of surface water source is complex due to the large number of water bodies in the country, their diverse geography, varying access and differing visitation characteristics. Therefore, the findings are proposed as triggers for new hypotheses that can eventually be confirmed through environmental monitoring strategies designed with the support and support of health and environmental authorities. Additionally, it is recommended that subsequent exploratory studies consider the isolation of FLA from sediments.

The extent of the KAP survey also limits the findings due to the number of respondents, but it allows for the establishment of a baseline that has not been available until now. Furthermore, although the questions were carefully formulated and reviewed prior to implementation, there are information biases inherent to the application of a survey that are difficult to control, so those that come from people should be considered primarily, such as memory bias, courtesy bias, affirmation bias and context bias, in response to external pressures from the social environment in which people live or were at the time of being surveyed.

## 5 Conclusion

In this study, thermotolerant FLA were isolated from 22 sampling sites; 7 of them resulted positive for *Naegleria*. Additionally, although some parameters were statistically associated with the presence of *Naegleria* in those sites, an epidemiological association was not demonstrated. This lack of a clear relationship of physicochemical parameters to the presence of *Naegleria* could be related to the variability of the measured parameters and the limited number of sites. However, the findings of the proposed environmental conditions´ survey represent a significant improvement in our knowledge of the presence of *Naegleria* and other Vahlkampfiid FLA capable of proliferating at high temperatures in natural non-thermal surface water reservoirs in Costa Rica and that could harbor other microorganisms of public health interest. These results also serve as a starting point for systematizing the monitoring of FLA in environments with close human contact.

From the KAP survey, 37.5% of participants had never heard about *N. fowleri*, and significant differences between the level of knowledge by gender and geographic origin were obtained. The KAP survey, a tool for elucidating what is known, believed and implemented in the context of the risk of *N. fowleri* infection, reinforces the importance and need for more and better health education, as a sufficient level of knowledge is not achieved, and the sample studied shows deficiencies in risk management and prevention. The gaps must be closed through effective communication and advertising strategies, utilizing both traditional media and social networks, in strategic cooperation with academia, healthcare providers, environmental health and water resource authorities, and other organizations related to public health.

## Data Availability

The raw data supporting the conclusions of this article will be made available by the authors, without undue reservation.
