# Supplementary material for: A socioenvironmental approach to the nosogenic potential of freshwaters with presence of thermotolerant free-living amoebae in Costa Rica
Source: Front Public Health. 2025 Oct 6;13:1675182. doi: 10.3389/fpubh.2025.1675182 (PMC12536000; doi:10.3389/fpubh.2025.1675182)
Supplement: Supplementary file 1 [file Data_Sheet_1.docx]

A socioenvironmental approach to the nosogenic potential of freshwaters with presence of thermotolerant free-living amoebae in Costa Rica

Supplementary Material

# Supplementary Tables

**Supplementary Table S1. Crude measure of association (OR) between independent variables and the presence of *Naegleria* in freshwater bodies.**

| **Matrix** | **Independent variable** | **OR** | **P** | **CI 95 %** | |
| --- | --- | --- | --- | --- | --- |
|  |  |  |  | **Lower limit** | **Upper limit** |
| Water | Temperature (°C) | 0.89 | 0.486 | 0.65 | 1.22 |
|  | pH | 8.49 | 0.104 | 0.65 | 111.60 |
|  | Dissolved solutes (ppm) | 0.95 | 0.178 | 0.89 | 1.02 |
|  | Electrical conductivity (µS/cm) | 0.98 | 0.167 | 0.94 | 1.01 |
|  | Dissolved oxygen (mg/dl) | 2.31 | 0.060 | 0.96 | 5.53 |
| Sediment | pH | 0.95 | 0.930 | 0.31 | 2.90 |
|  | Ca (cmol(+)/l) | 0.97 | 0.836 | 0.74 | 1.28 |
|  | Mg (cmol(+)/l) | 0.88 | 0.630 | 0.53 | 1.47 |
|  | K (cmol(+)/l) | 0.00002 | 0.088 | 1.3x10^-8 | 3.54 |
|  | P (cmol(+)/l) | 0.82 | 0.527 | 0.44 | 1.52 |
|  | Zn (mg/l) | 0.58 | 0.188 | 0.26 | 1.31 |
|  | Cu (mg/l) | 0.42 | 0.062 | 0.17 | 1.04 |
|  | Fe (mg/l) | 0.98 | 0.359 | 0.94 | 1.02 |
|  | Mn (mg/l) | 0.94 | 0.512 | 0.78 | 1.13 |
|  | Electrical conductivity (mS/cm) | 0.0002 | 0.257 | 1.1x10^-10 | 454.30 |
|  | % Sand | 1.35 | 0.140 | 0.91 | 2.01 |
|  | % Silt | 0.88 | 0.387 | 0.66 | 1.17 |
|  | % Clay | 0.60 | 0.080 | 0.34 | 1.06 |

**Supplementary Table S2. Frequency of the type of response offered for each attitude and practice item, concerning visiting freshwater bodies for recreation and the risk of infection by *Naegleria fowleri*.**

| **Value on the Likert Scale** | **Answer option for each statement** | **ATTITUDES** | | | | **Value on the Likert Scale** | **Answer option for each statement** | **PRACTICES** | | |
| --- | --- | --- | --- | --- | --- | --- | --- | --- | --- | --- |
|  |  | Infection is a major health problem | There is concern about acquiring the infection | Attention is paid to information related to this infection | Go to the health center if experiencing symptoms related to the infection |  |  | Wear a nose clip when doing water activities | Avoid submerging or diving when not having a nose clip | Recommend the previous prevention measures to others |
| **5** | **Totally disagree** | 5 | 11 | 5 | 9 | **5** | **Never** | 58 | 29 | 36 |
| **4** | **Disagree** | 4 | 5 | 6 | 10 | **4** | **Most of the time no** | 4 | 3 | 5 |
| **3** | **Neither agree nor disagree** | 11 | 11 | 13 | 15 | **3** | **Sometimes yes, sometimes no** | 5 | 10 | 13 |
| **2** | **Agree** | 16 | 11 | 17 | 11 | **2** | **Most of the time yes** | 2 | 14 | 5 |
| **1** | **Totally agree** | 36 | 34 | 31 | 27 | **1** | **Always** | 3 | 16 | 13 |
| **Total responses** | | 72 | | | | | | | | |
